# Supplementary material for: Discovery of levodopa-induced dyskinesia-associated genes using genomic studies in patients and Drosophila behavioral analyses
Source: Commun Biol. 2022 Aug 25;5:872. doi: 10.1038/s42003-022-03830-x (PMC9411113; doi:10.1038/s42003-022-03830-x)
Supplement: Supplementary file 2 — Supplementary Information [file 42003_2022_3830_MOESM2_ESM.pdf]

Supplementary Information

Supplementary Note 1: MATLAB code for modeling of fly AIM scores

```
window_size = 250;

threshold = 0.4;

aim = 0;


for i=(floor(window_size/2)+1):(length(trx.velmag_ctr)-floor(window_size/2))

    if ((log10(trx.velmag_ctr(i)/mean(trx.velmag_ctr((i-
floor(window_size/2)):(i+floor(window_size/2)))))) > threshold)

        aim = aim + 1;

    end

end


fprintf('Fly speed : %f\n', mean(trx.velmag_ctr))

fprintf('Fly absyaw : %f\n', mean(trx.absyaw))

fprintf('Fly pause : %f\n', mean(trx.corisonfly))

fprintf('Fly Aim score : %f\n', aim)
```

Supplementary Fig. 1: Survival rate upon prolonged 10 mM L-DOPA diets.

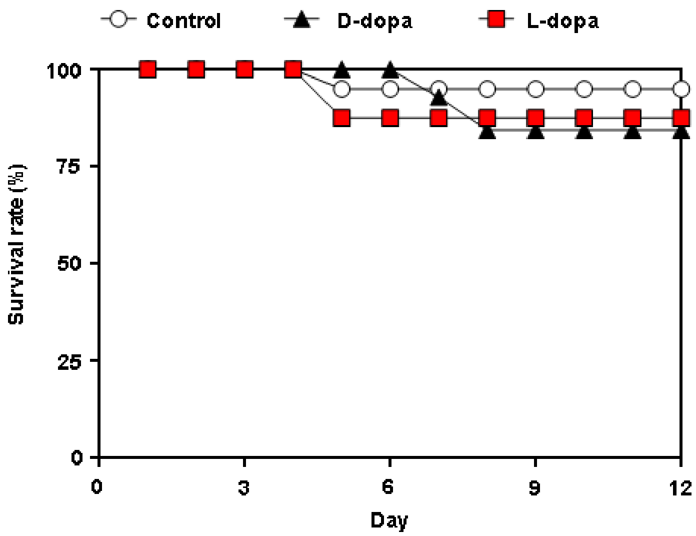

Comparison of the survival rate of the flies with indicated diet. N=7. Statistical significance was analyzed by log-rank (Mantel-Cox) test. The P-value is 0.7532, which is statistically not significant ($p>0.05$).

Supplementary Fig. 2: Movements comparisons of PD model flies upon acute L-DOPA treatments.

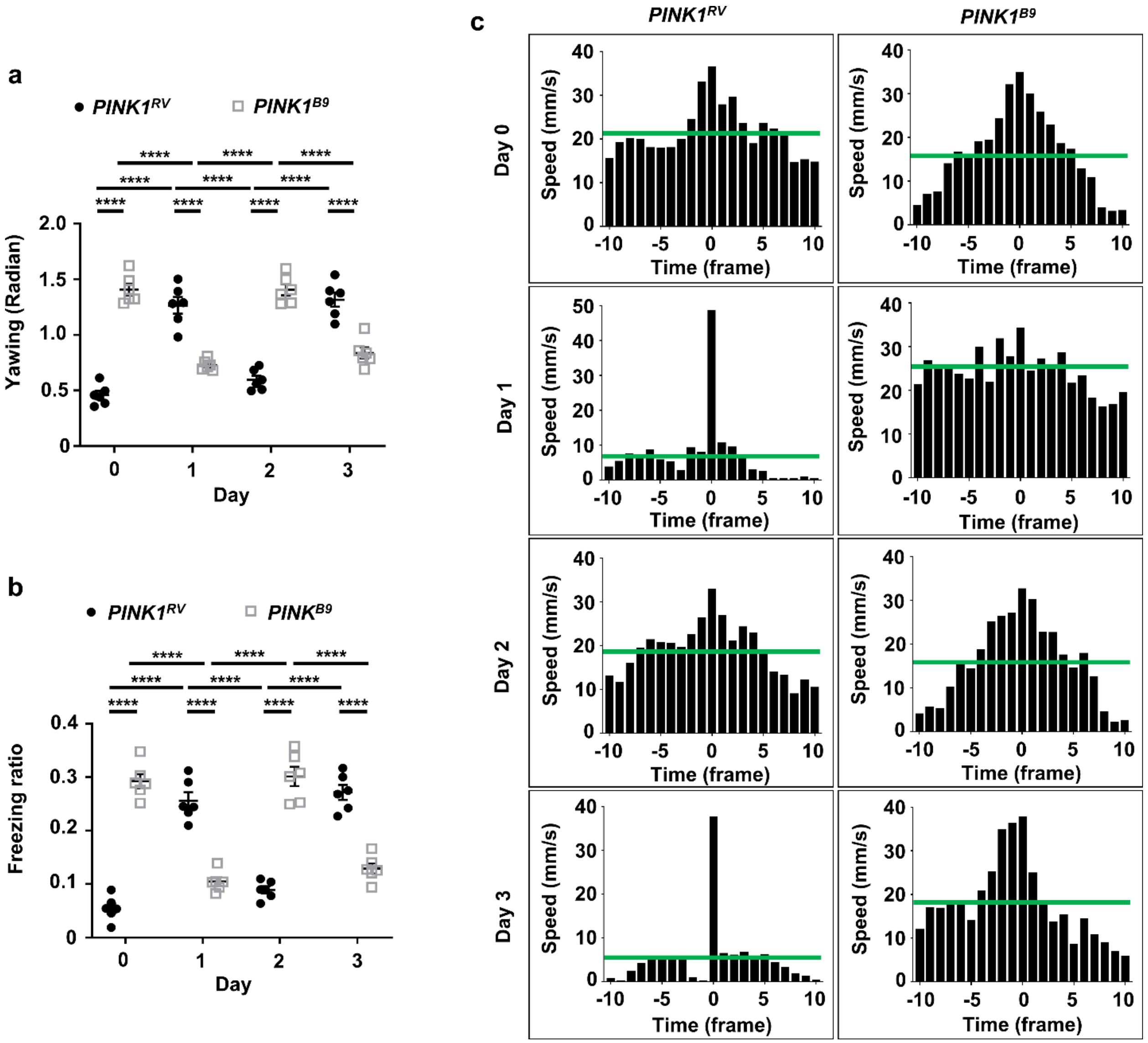

a, b Comparisons of the quantified yawing **a** and fraction of freezing **b** of control flies and PD model flies upon acute L-DOPA diets. N=6. ****, $p<0.0001$; ***, $p<0.001$ by two-way ANOVA Tukey's multiple comparison test. Data are presented as means ± SEM. $p<0.05$ was considered statistically significant. **c** Comparisons of the instantaneous speed 10 frames before and after the

peak of speed by the control and PD model flies upon acute L-DOPA diets intercalated with normal diets and acute L-DOPA diet. Mean speeds within the entire time period (21 frames) are denoted by green lines.

Supplementary Fig. 3: Movements comparisons of PD model flies upon chronic L-DOPA treatments.

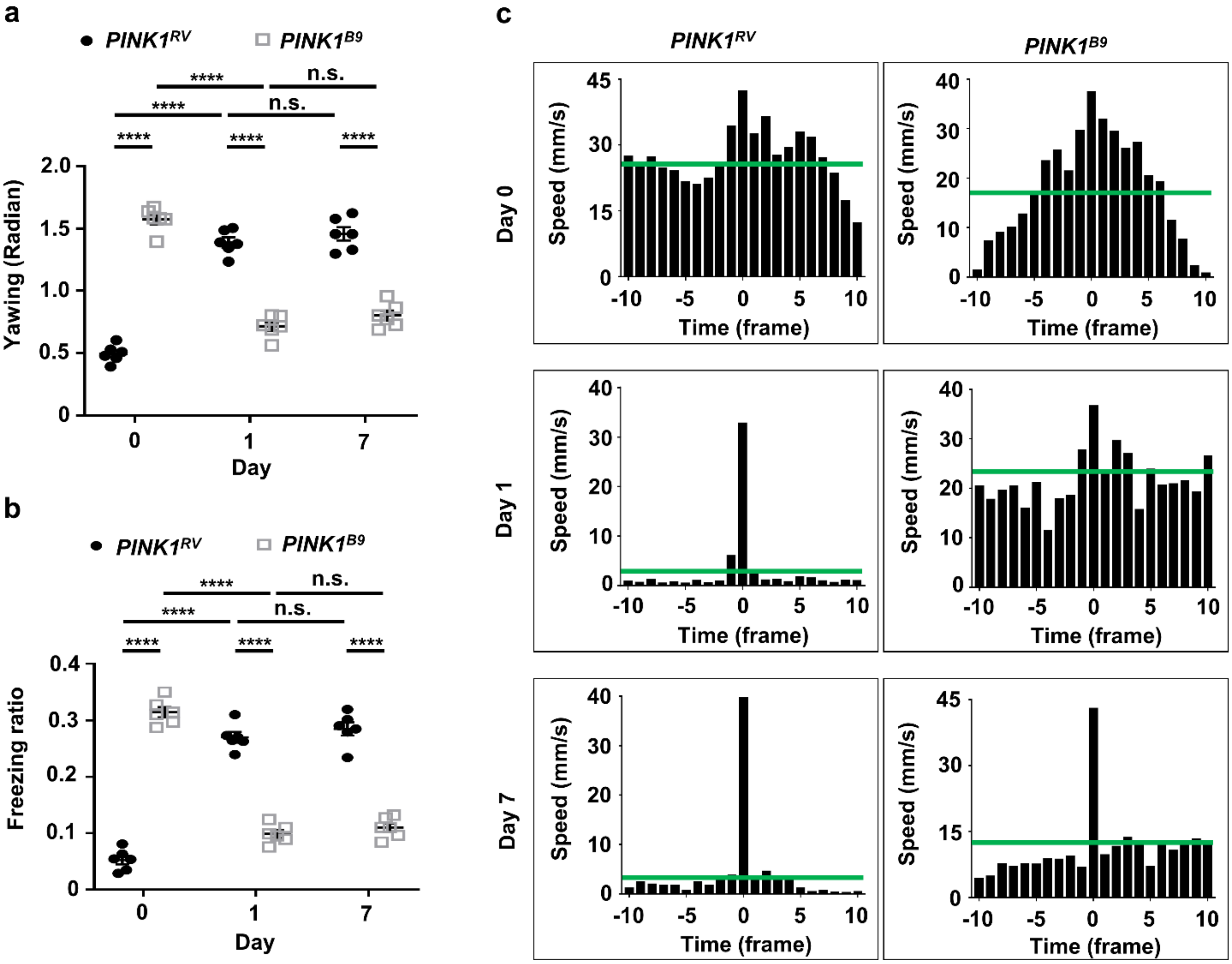

a, b Comparisons of the quantified yawing **a** and fraction of freezing **b** of the control and PD model flies upon chronic L-DOPA diet. N=6. ****, $p<0.0001$; **, $p<0.01$; n.s., not significant ($p>0.05$) by two-way ANOVA Tukey's multiple comparison test. Data are presented as means ± SEM. $p<0.05$ was considered statistically significant. **c** Comparisons of the instantaneous speed 10 frames before and after the peak of speed by the control and PD model flies upon acute L-DOPA diets intercalated with normal diets and chronic L-DOPA diet. Mean speeds within the

entire time period (21 frames) are denoted by green lines.

Supplementary Fig. 4: Genome-wide association study (GWAS) on PD patients diagnosed with L-DOPA induced dyskinesia (LID).

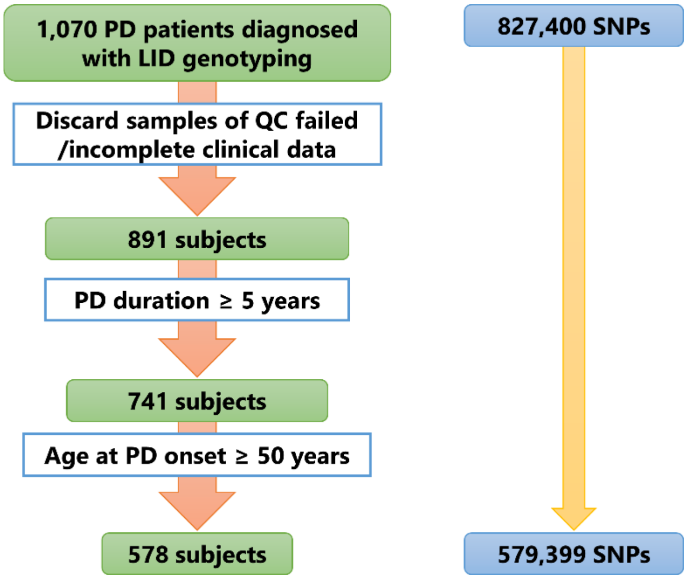

A flowchart illustrating sequential criteria in selection of PD patient subjects and their genotyping data set for GWAS.

Supplementary Fig. 5: Gene ontology (GO) enrichment and KEGG pathway analysis of LID-associated genes (LAGs).

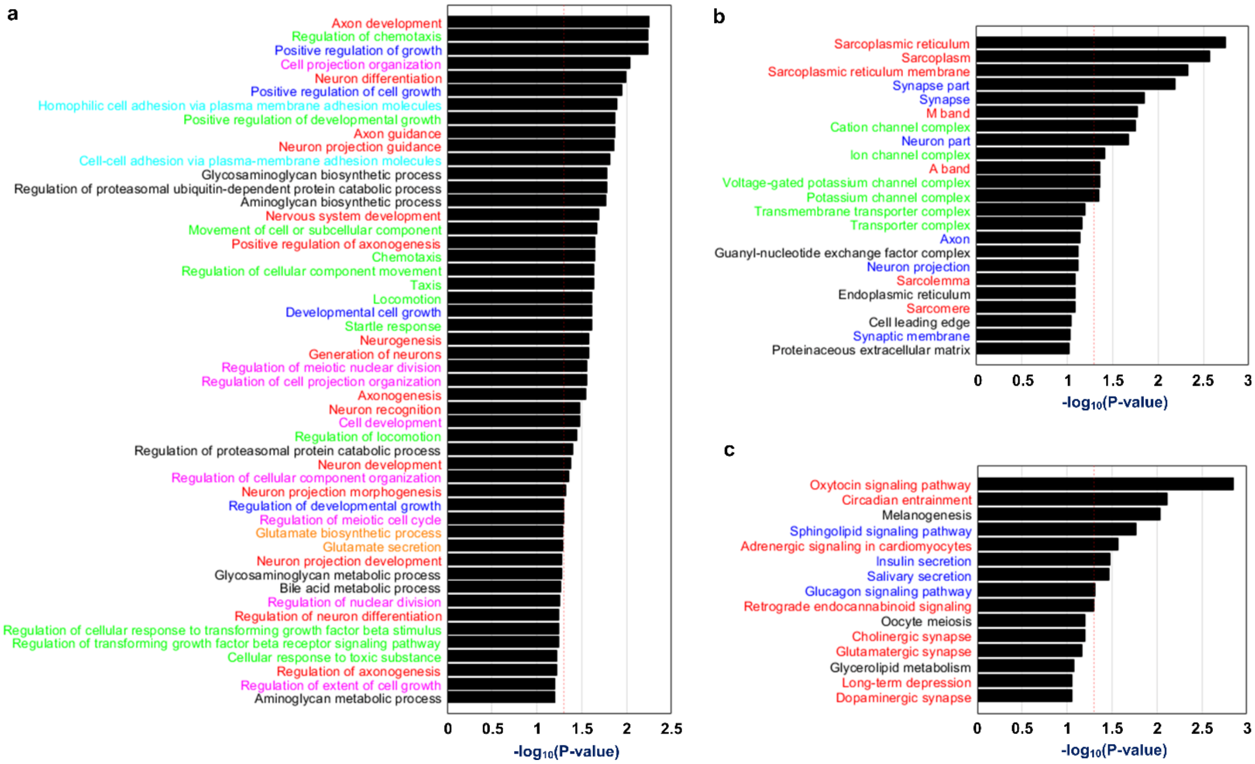

a, b GO enrichment analyses on LAGs. From highly to lowly (top to bottom) enriched Biological processes involved by LAGs. Reds indicate Nervous system development category terms. Greens indicate Response to stimulus/ Locomotion category terms. Magentas indicate Cellular component organization/ Biogenesis category terms. Blues indicate Developmental growth category terms. Cyans indicate Adhesion category terms. Oranges indicate Nervous system process category terms. **b** From highly to lowly enriched Cellular component involved by LAGs. Reds indicate Muscle cell category terms. Greens text indicate Transporter complex category terms. Blues indicate neural cell category terms. **c** Mapped pathways by KEGG pathway analysis. Reds indicate Nervous system category pathway. Blues indicate Endocrine

system category terms.

Supplementary Fig. 6: Networks used in network analysis and their components.

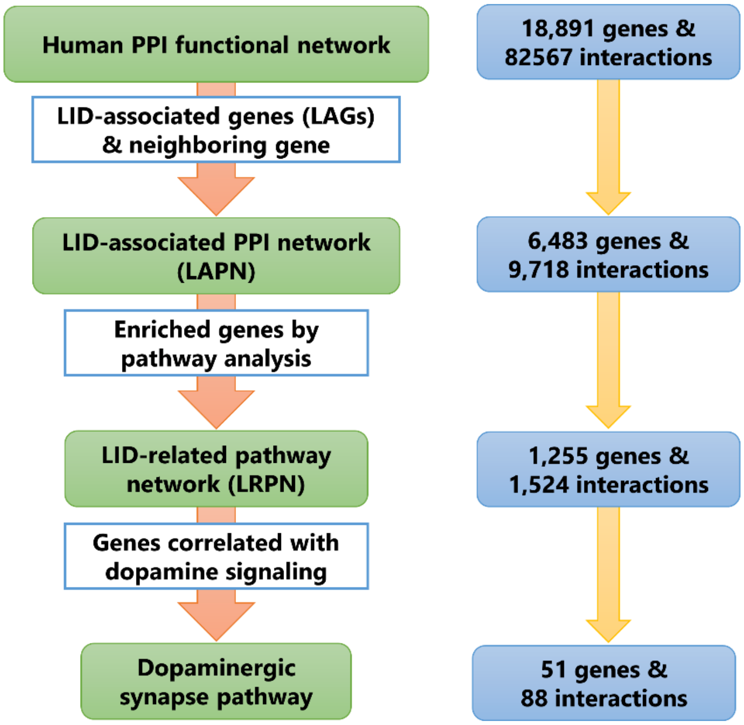

A flowchart illustrating composition methods and elements of networks for network analysis.

Supplementary Fig. 7: LID-related pathway network (LRPN), LID-associated PPI network (LAPN)'s subnetwork.

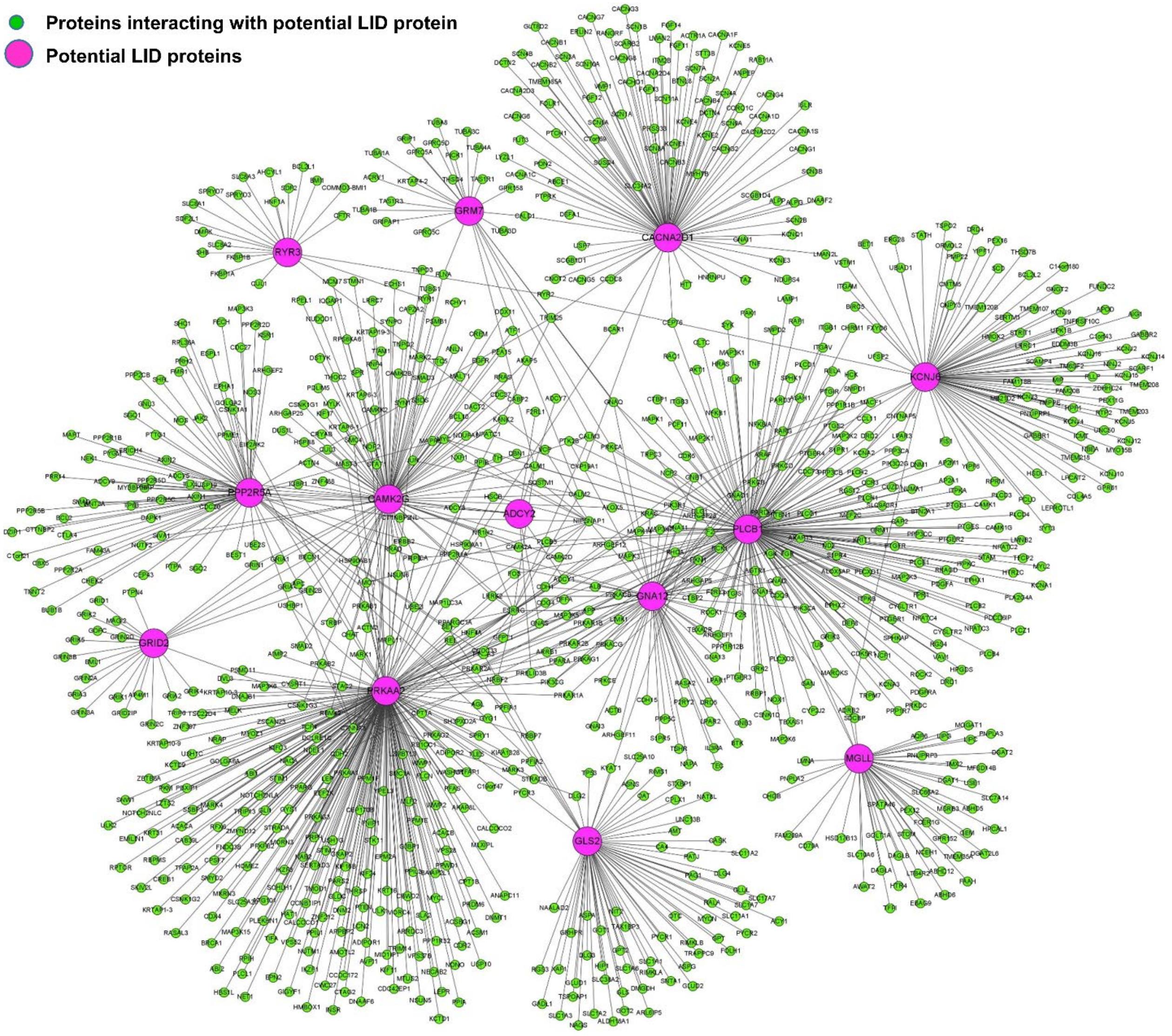

The LRPN was constructed by pathway analysis with the genes constituting LAPN. Margenta nodes indicate potential LID proteins encoded by LAGs. Green nodes indicate proteins interacting with the potential LID protein that serves as a neighboring node.

Supplementary Fig. 8: A subnetwork reconstructed by removing terminal nodes from LAPN.

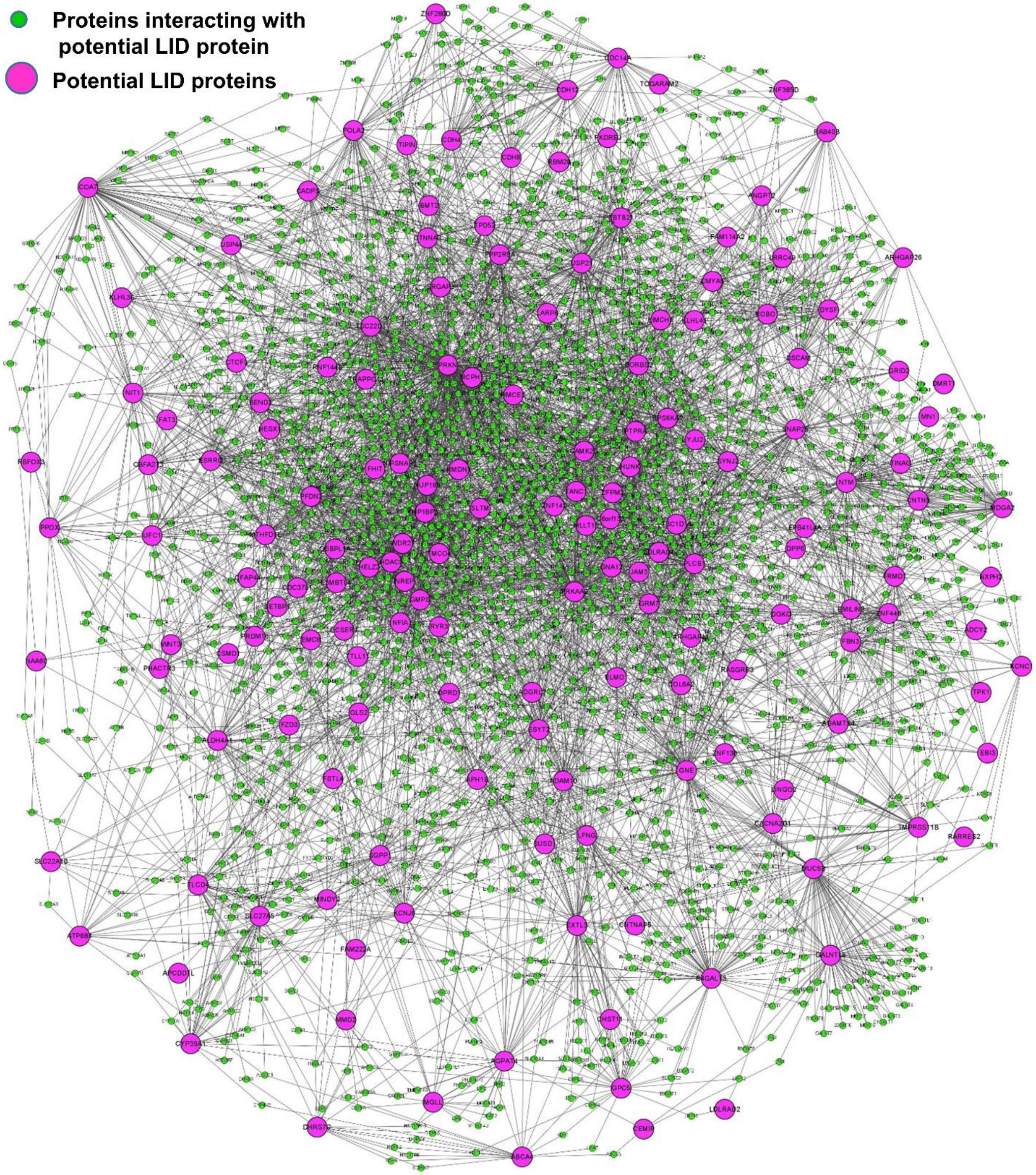

The terminal nodes were removed from LAPN to build this subnetwork. Margenta nodes indicate

potential LID proteins encoded by LAGs. Green nodes indicate proteins interacting with the potential LID protein that serves as a neighboring node, not termianl nodes.

Supplementary Fig. 9: Phylogenetic analyses of ADCY members in human and flies.

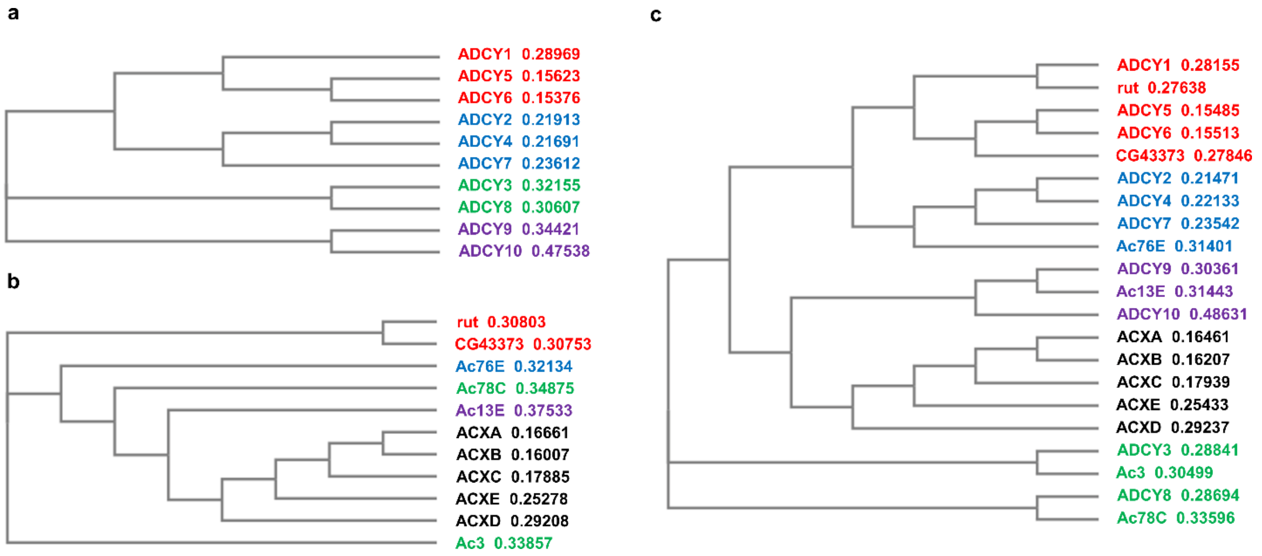

a Phylogenetic tree of the ADCY family members in human. Reds indicate group composed of *ADCY1/5/6*. Blues indicate group composed of *ADCY2/4/7*. Greens indicate group composed of *ADCY3/8*. Purples indicate group composed of *ADCY9/10*. **b** Phylogenetic tree of the ADCY family members in *Drosophila*. Reds indicate group composed of *dADCY1/5*. Blues indicate group composed of *dADCY2*. Greens indicate group composed of *dADCY3/8*. Purples indicate group composed of *dADCY9*. **c** Phylogenetic relationship between human ADCY family and *Drosophila* members. The numbers represent the number of differences between sequences. Reds indicate group composed of human and *Drosophila ADCY1/5/6*. Blues indicate group composed of human and *Drosophila ADCY2/4/7*. Greens indicate group3 composed of human and *Drosophila ADCY3/8*. Purples indicate group4 composed of human and *Drosophila ADCY9/10*.

Term	P-value	Genes
**hsa04921:Oxytocin signaling pathway**	**0.001411**	***RYR3, CACNA2D1, PRKAA2, KCNJ2, ADCY2, PLCB1, CAMK2G***
**hsa04713:Circadian entrainment**	**0.007713**	***RYR3, KCNJ2, ADCY2, PLCB1, CAMK2G***
**hsa04916:Melanogenesis**	**0.009215**	***WNT3, FDZ3, ADCY2, PLCB1, CAMK2G***
**hsa04071:Sphingolipid signaling pathway**	**0.017094**	***GNA12, OPRD1, PPP2R5A, SGPP1, PLCB1***
**hsa04261:Adrenergic signaling in cardiomyocytes**	**0.026988**	***CACNA2D1, ADCY2, PPP2R5A, PLCB1, CAMK2G***
**hsa04911:Insulin secretion**	**0.033025**	***ADCY2, PLCB1, SNAP25, CAMK2G***
**hsa04970:Salivary secretion**	**0.034022**	***RYR3, ADCY2, PLCB1, MUC5B***
**hsa04922:Glucagon signaling pathway**	**0.048391**	***PRKAA2, ADCY2, PLCB1, CAMK2G***
**hsa04723:Retrograde endocannabinoid signaling**	**0.050829**	***KCNJ2, ADCY2, MGLL, PLCB1***
**hsa04114:Oocyte meiosis**	**0.063897**	***6196, ADCY2, PPP2R5A, CAMK2G***
**hsa04725:Cholinergic synapse**	**0.063897**	***KCNJ2, ADCY2, PLCB1, CAMK2G***
**hsa04724:Glutamatergic synapse**	**0.068094**	***GLS2, ADCY2, GRM7, PLCB1***
**hsa00561:Glycerolipid metabolism**	**0.083063**	***AGPAT4, DGKQ, MGLL***
**hsa04730:Long-term depression**	**0.088028**	***GNA12, GRID2, PLCB1***
**hsa04728:Dopaminergic synapse**	**0.089277**	***KCNJ2, PPP2R5A, PLCB1, CAMK2G***

Supplementary Table 1: Pathways and their involved LAGs analyzed using KEGG pathway analysis. Pathways mapped by KEGG pathway analysis using LAGs. Enriched pathways (left) are arranged in order of low to high p-values (top to bottom) and their LAGs are listed (right).

Number	Fruit fly gene	Human gene	Stock ID	Stock source
**#1**	***CG14619***	***USP21***	**104382**	**VDRC**
**#2**	***IR25a***	***GRID2***	**43985**	**BDSC**
**#3**	***Plc21c***	***PLCB1***	**33719**	**BDSC**
**#4**	***wdb***	***PPP2R5A***	**38901**	**BDSC**
**#5**	***GluRIB***	***GRID2***	**40908**	**BDSC**
**#6**	***S6KII***	***RPS6KA2***	**61202**	**BDSC**
**#7**	***CG33144***	***RNF144B***	**64033**	**BDSC**
**#8**	***cdc14***	***CDC14A***	**103627**	**VDRC**
**#9**	***ex***	***FRMD1***	**34968**	**BDSC**
**#10**	***NitFhit***	***NIT1***	**55285**	**BDSC**
**#11**	***CG34124***	***CFAP44***	**105659**	**VDRC**
**#12**	***Shaw***	***KCNC1***	**28346**	**BDSC**
**#13**	***Ppox***	***PPOX***	**51777**	**BDSC**
**#14**	***CG42708***	***GLS2***	**62216**	**BDSC**
**#15**	***esyt2***	***ESYT2***	**28419**	**VDRC**
**#16**	***wrd***	***PPP2R5A***	**30512**	**BDSC**
**#17**	***Coa7***	***COA7***	**60349**	**BDSC**
**#18**	***CG10336***	***TIPIN***	**60378**	**BDSC**
**#19**	***cta***	***GNA12***	**51848**	**BDSC**
**#20**	***dtn***	***TMEM132C***	**32487**	**BDSC**
**#21**	***S6KL***	***RPS6KA2***	**35178**	**BDSC**
**#22**	***dADCY2***	***ADCY2***	**106232**	**VDRC**
**#23**	***shawl***	***KCNC1***	**25819**	**BDSC**

Supplementary Table 2: List of the fly RNAi lines used for LID-related behavioral study. The RNAi fly lines representing the 23 *Drosophila* genes homologous to the 19 LID-related human genes screened from LID patient SNPs.

Human gene	Fruit fly gene	Similarity (%)
ADCY1	***rut***	**56.88**
ADCY2	***Ac76E***	**53.83**
ADCY3	***Ac3***	**51.20**
ADCY5	***CG43373***	**59.80**
ADCY8	***Ac78C***	**52.28**
ADCY9	***Ac13E***	**53.03**

Supplementary Table 3: Summary of the fly *ADCY* genes. Orthologous relationships between human ADCY genes and *Drosophila* ADCY genes.
