# Supplementary material for: Discovery of levodopa-induced dyskinesia-associated genes using genomic studies in patients and Drosophila behavioral analyses
Source: Commun Biol. 2022 Aug 25;5:872. doi: 10.1038/s42003-022-03830-x (PMC9411113; doi:10.1038/s42003-022-03830-x)
Supplement: Supplementary file 3 — Description of Additional Supplementary Files [file 42003_2022_3830_MOESM3_ESM.pdf]

# Description of Additional Supplementary Files

**File name:** Supplementary Data 1
**Description:** Numerical source data for graphs.
