# Supplementary material for: Discovery of levodopa-induced dyskinesia-associated genes using genomic studies in patients and Drosophila behavioral analyses
Source: Commun Biol. 2022 Aug 25;5:872. doi: 10.1038/s42003-022-03830-x (PMC9411113; doi:10.1038/s42003-022-03830-x)
Supplement: Supplementary file 5 — Reporting Summary [file 42003_2022_3830_MOESM5_ESM.pdf]

nature portfolio

Corresponding author(s): Jongkyeong Chung

Last updated by author(s): Jul 27, 2022

Reporting Summary

Nature Portfolio wishes to improve the reproducibility of the work that we publish. This form provides structure for consistency and transparency in reporting. For further information on Nature Portfolio policies, see our Editorial Policies and the Editorial Policy Checklist.

Statistics

For all statistical analyses, confirm that the following items are present in the figure legend, table legend, main text, or Methods section.

n/a	Confirmed	
☐	☒	The exact sample size (n) for each experimental group/condition, given as a discrete number and unit of measurement
☐	☒	A statement on whether measurements were taken from distinct samples or whether the same sample was measured repeatedly
☐	☒	The statistical test(s) used AND whether they are one- or two-sided<br>*Only common tests should be described solely by name; describe more complex techniques in the Methods section.*
☒	☐	A description of all covariates tested
☐	☒	A description of any assumptions or corrections, such as tests of normality and adjustment for multiple comparisons
☐	☒	A full description of the statistical parameters including central tendency (e.g. means) or other basic estimates (e.g. regression coefficient) AND variation (e.g. standard deviation) or associated estimates of uncertainty (e.g. confidence intervals)
☐	☒	For null hypothesis testing, the test statistic (e.g. F, t, r) with confidence intervals, effect sizes, degrees of freedom and P value noted<br>*Give P values as exact values whenever suitable.*
☒	☐	For Bayesian analysis, information on the choice of priors and Markov chain Monte Carlo settings
☒	☐	For hierarchical and complex designs, identification of the appropriate level for tests and full reporting of outcomes
☒	☐	Estimates of effect sizes (e.g. Cohen's d, Pearson's r), indicating how they were calculated

Our web collection on statistics for biologists contains articles on many of the points above.

Software and code

Policy information about availability of computer code

Data collection	Fly video tracking was done using the Ctrax fly tracking program for reliable object detection (http://ctrax.sourceforge.net/).
Data analysis	The fly movements were analyzed using 'Behavioral Microarray MATLAB toolbox' available from Ctrax website (http://ctrax.sourceforge.net/). Statistical analyses were calculated with GraphPad Prism (ver. 7) and R Statistics (ver. 4.0.4).

For manuscripts utilizing custom algorithms or software that are central to the research but not yet described in published literature, software must be made available to editors and reviewers. We strongly encourage code deposition in a community repository (e.g. GitHub). See the Nature Portfolio guidelines for submitting code & software for further information.

Data

Policy information about availability of data

All manuscripts must include a data availability statement. This statement should provide the following information, where applicable:

- Accession codes, unique identifiers, or web links for publicly available datasets
- A description of any restrictions on data availability
- For clinical datasets or third party data, please ensure that the statement adheres to our policy

The authors declare that all data supporting the findings of this study are available within the article and its supplementary information files. The source data that support the findings of this study are available in Mendeley Data with the identifier data DOI http://doi.org/10.17632/48rhgh28xn.1. The human genomic data that support the findings of this study are available in dbSNP, https://www.ncbi.nlm.nih.gov/SNP/snp_viewBatch.cgi?sbid=1063124.

Field-specific reporting

Please select the one below that is the best fit for your research. If you are not sure, read the appropriate sections before making your selection.

☒ Life sciences ☐ Behavioural & social sciences ☐ Ecological, evolutionary & environmental sciences

For a reference copy of the document with all sections, see nature.com/documents/nr-reporting-summary-flat.pdf

Life sciences study design

All studies must disclose on these points even when the disclosure is negative.

Sample size	The sample size in a experiment was 6-12, which is large enough to handle fly behavioral experiments. The sample size was determined based on published studies in this field or our previously published studies. No statistics were used to predetermine the sample size.
Data exclusions	None
Replication	3-4 duplicate experiments were performed for each experiment. All attempts at replication were successful.
Randomization	The flies used in the experiment were randomly selected.
Blinding	The investigator was not blinded for the allocation of behavior groups for the experiment. However, since flies were always randomly selected and used in all experiments, the investigator was unaware of the outcome of experiment.

Reporting for specific materials, systems and methods

We require information from authors about some types of materials, experimental systems and methods used in many studies. Here, indicate whether each material, system or method listed is relevant to your study. If you are not sure if a list item applies to your research, read the appropriate section before selecting a response.

Materials & experimental systems

n/a	Involved in the study	
☒	☐	Antibodies
☒	☐	Eukaryotic cell lines
☒	☐	Palaeontology and archaeology
☐	☒	Animals and other organisms
☒	☐	Human research participants
☒	☐	Clinical data
☒	☐	Dual use research of concern

Methods

n/a	Involved in the study	
☒	☐	ChIP-seq
☒	☐	Flow cytometry
☒	☐	MRI-based neuroimaging

Animals and other organisms

Policy information about studies involving animals; ARRIVE guidelines recommended for reporting animal research

Laboratory animals	Newly-born fruit flies aged for 1-2 days were collected and used. Female flies were not used due to their egg-laying behaviors. Drosophila stocks were purchased from the Bloomington Drosophila Stock Center (BDSC), and the Vienna Drosophila Resource Center (VDRC). Information on all Drosophila strains used in this study was provided in the Methods section.
Wild animals	None
Field-collected samples	None
Ethics oversight	None

Note that full information on the approval of the study protocol must also be provided in the manuscript.
